# Preliminary treatment results of proton beam therapy with chemoradiotherapy for stage I–III esophageal cancer

**DOI:** 10.1002/cam4.607

**Published:** 2016-01-24

**Authors:** Akinori Takada, Tatsuya Nakamura, Kanako Takayama, Chiyoko Makita, Motohisa Suzuki, Yusuke Azami, Takahiro Kato, Iwao Tsukiyama, Masato Hareyama, Yasuhiro Kikuchi, Takashi Daimon, Yutaka Toyomasu, Noriko Ii, Yoshihito Nomoto, Hajime Sakuma, Nobukazu Fuwa

**Affiliations:** ^1^ Department of Radiation OncologyMie University HospitalTsuMieJapan; ^2^ Southern Tohoku Proton Therapy CenterKoriyamaFukushimaJapan; ^3^ Department of Dentistry/oral surgeryGraduate School of MedicineYokohama City UniversityFukuura, Kanazawa‐kuYokohamaJapan; ^4^ Department of Radiation OncologyAichi Cancer Center HospitalChikusakuNagoyaJapan; ^5^ Division of BiostatisticsHyogo College of MedicineNishinomiyaHyogoJapan; ^6^ Hyogo Ion Beam Medical CenterShingu, TatsunoHyogoJapan

**Keywords:** Alternating chemoradiotherapy, cardiopulmonary complication, esophageal cancer, late toxicity, proton beam therapy

## Abstract

The effect of proton beam therapy (PBT) on various cancers is controversial. We aimed to evaluate the efficacy and safety of PBT with alternating chemoradiotherapy (ACRT) for patients with stage I–III esophageal cancer. Two cycles of systemic chemotherapy with a continuous infusion of 5‐fluorouracil (5‐FU) on days 1–5 and a 5h infusion of nedaplatin (NDP) on day 6 were accompanied by thoracic irradiation using X‐ray therapy and PBT. During the first half of the treatment, X‐rays were delivered to the prophylactic area. During the second half of the treatment, proton beams were used to irradiate the involved field. To reduce the dose of cardiac irradiation, proton beams were delivered with posterior and posterior oblique angles. Between January 2009 and December 2012, 47 patients were enrolled in this study. The median follow‐up duration was 29 months for all patients and 40 months for survivors. The 3 year overall survival rate, progression‐free survival rate, and local control rate were 59.2%, 56.3%, and 69.8%, respectively. With respect to grade 3–4 late toxicities, there were no pleural or pericardial effusions, but two patients (4.3%) had esophageal stenosis, one patient (2.1%) had fistula, and two patients (4.3%) developed radiation pneumonitis. PBT with ACRT might have the potential to reduce the risk of cardiac damage and might become one of the primary methods of esophageal cancer treatment.

## Introduction

Although the standard treatment for esophageal cancer is surgery, the frequency of serious complications and the death rate related to surgery are higher than those of other digestive organ cancers 1. Previous reports have shown that the treatment‐related mortality rate from thoracic esophageal cancer surgery is 3.8–7.9% 2, 3. Radiotherapy alone has been used to treat elderly patients, those with medically inoperable cancer, those with generalized poor functional capacity, and patients who decline surgery. However, the treatment outcomes are poor, with 5‐year survival rates ranging from 0% to 9% 4, 5. With the development of chemoradiotherapy (CRT) for esophageal cancer, treatment outcomes have significantly improved, and this treatment approach is now acknowledged as a standard treatment for esophageal cancer 6. Because it has the apparent advantage of being an esophagus‐conserving therapy, which may lead to a better quality of life for patients, definitive CRT is a treatment option for patients with any stage disease. In particular, concurrent CRT (CCRT) has become a standard alternative treatment to surgery 6. However, serious late adverse effects such as radiation pneumonitis, pericardial effusion, and other complications after CRT due to high doses of radiation to the lung and heart are a critical issue. Rates of grade 3 or higher pericardial effusion and pneumonitis as late adverse events have been found to be 9–10.3% and 3.8–4.0%, respectively 6, 7, 8. In a series of 78 patients treated with CRT, Ishikura et al. reported that two (2.6%) patients died of myocardial infarction and eight (10.2%) died of pericardial or pleural effusion 8. CCRT for esophageal cancer might decrease the therapeutic response due to the large irradiation field. This, in turn, could lead to severe esophagitis, necessitating a break in treatment. We therefore adopted a strategy of alternating CRT (ACRT) for CCRT.

Local recurrence after CCRT, which occurs at a rate of approximately 50%, is also a problem with this therapy, and dose escalation has been proposed to reduce this recurrence 6. Regarding radiation dose escalation for patients with esophageal cancer, Minsky et al. reported that there was no significant difference in survival or locoregional control (LC) between the standard‐dose arm and the high‐dose arm. The lack of benefit from high‐dose radiation therapy was presumed to be due in part to treatment breaks necessitated by toxicity 9. Suh et al. reported that radiation doses of 60 Gy or higher with concurrent chemotherapy improved both LC and progression‐free survival (PFS) without a significant increase in treatment‐related toxicity in patients with stages II–III esophageal cancer 10. Additionally, not for esophageal cancer, but for larynx cancer and pharynx cancer, the excellent outcomes were reported using fractional (2.2–2.5 Gy) dose escalation to treat them 11, 12.

In contrast to conventional radiation therapy, proton beam therapy (PBT) takes advantage of Bragg peak properties and can deliver a high radiation dose to the tumor while largely sparing normal tissue. We assumed that a higher radiation dose might result in an improvement in local control, and we adopted an enhanced dosage of 2.2 GyE by PBT to the primary site and lymph node metastasis as boost irradiation.

PBT has shown excellent results for head and neck cancer and hepatic cancer 13, 14. However, there have been few studies on the use of PBT for esophageal cancer, and its clinical significance in treating this cancer remains unknown 15, 16. In January 2009, we began treating esophageal cancer with PBT to achieve a higher antitumor effect without severe heart and lung complications. In this study, we present an analysis of the preliminary treatment results and a verification of the clinical importance of PBT for esophageal cancer. The treatment method and procedure were approved by the local institutional review board. All procedures followed were in accordance with the Helsinki Declaration of 1975.

## Materials and Methods

### Eligibility criteria

This study was based on a retrospective analysis of a prospectively collected database. Patients with esophageal cancer who met the following criteria were enrolled in this study: presence of pathologically confirmed esophageal cancer; clinical stage I–III cancer according to the Union for International Cancer Control (UICC) 2009; performance status (PS) 0–2 according to the Eastern Cooperative Oncology Group (ECOG) criteria; age 20–80 years; absence of tracheoesophageal fistulas; sufficient bone marrow function (white blood cell [WBC] count >3500/mm^2^ and platelet count >100,000/mm^2^); absence of abnormalities in the liver, kidneys, heart, and lungs (for renal function, a 24‐h creatinine clearance ≥60 mL/min); untreated esophageal cancer; absence of active double cancer at the beginning of treatment; and written informed consent.

The extent of the primary esophageal lesion was evaluated by contrast enhancement‐CT (CE‐CT), CT/PET with 2‐[fluorine‐18]‐fluoro‐2‐deoxy‐d‐glucose (FDG‐PET/CT), a barium study, and fiberoptic endoscopy. Metastasis to the lymph nodes was evaluated by CT or PET‐CT and palpation. The presence or absence of distant metastasis was investigated by chest radiography, CT, and PET‐CT.

### Treatment procedure summary

**Figure 1:**
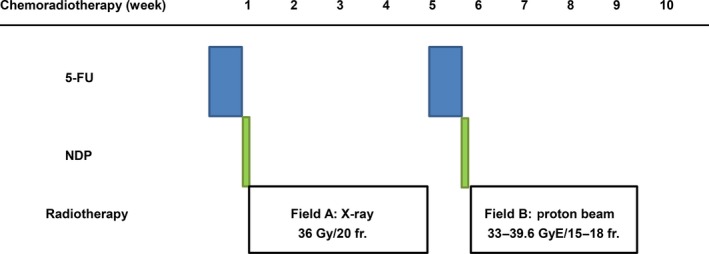
Study design of alternating chemoradiotherapy with proton beam therapy in patients with esophageal cancer.

The treatment scheme for ACRT is shown in Figure 1. First, patients received an initial course of chemotherapy followed by conventional wide‐field X‐ray therapy (XRT) that included the prophylactic area. Following XRT, patients received a second course of chemotherapy followed by PBT.

### Radiation therapy

Patients received XRT five times a week for 4 weeks beginning 1–3 days after each course of chemotherapy. XRT was performed by delivering 1.8 Gy fractions of photon beams with a 6–10 MV linear accelerator. Using the anterior–posterior opposing portal irradiation method, 36 Gy in 20 fractions was delivered between the supraclavicular fossa and the perigastric lymph nodes (Fig. 1, Field A). A prophylactic nodal area was defined between the bilateral supraclavicular fossae and superior mediastinal lymph nodes for carcinoma of the upper thoracic esophagus, between the bilateral supraclavicular fossa and perigastric lymph nodes for carcinoma of the middle esophagus, and between the mediastinal and perigastric lymph nodes for carcinoma of the lower and abdominal esophagus. For the second half of radiotherapy, PBT (Xio‐M; CMS Japan, Tokyo, Japan; Mitsubishi Electric Corporation, Kobe, Japan) was applied, wherein concentrated dosage was delivered to the primary tumor and lymph node metastasis (Fig. 1, Field B). The total dose of PBT was 33–39.6 GyE per 15–18 fractions. The proton dose is given in GyE using a relative biological effectiveness value of 1.1. PBT was performed five times a week by irradiating with 2.2 GyE fractions of 150 MeV or 210 MeV proton beams. Irradiation to the esophagus and the metastatic lymph nodes was performed using the dual portal broad beam (passive) method using multileaf collimators (Fig. 1, Field B). The site of the primary proton treatment field was determined as follows: During endoscopy prior to therapy, the lesion of the primary site to be irradiated with proton beams was marked with metal clips at the cranial and caudal ends 10 mm away from the area that had not been dyed with Lugol's solution. For the cases in which a gastrointestinal fiberscope (GIF) was unable to pass through the esophagus, the markers were implanted only at the cranial boundary of the primary tumor, and the caudal boundary was determined using imaging findings. A three‐dimensional treatment planning system for PBT was used.

**Figure 2:**
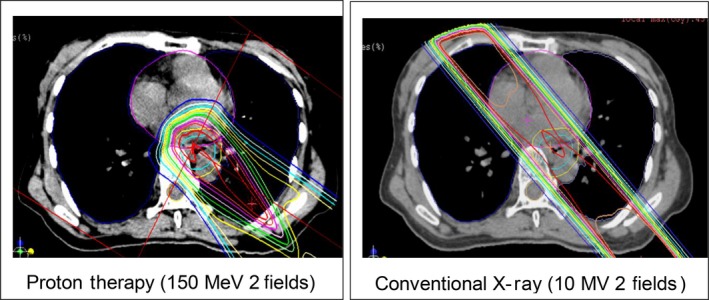
Dose distribution (proton beam therapy vs. conventional radiotherapy).

Regarding the PBT field, the gross tumor volume (GTV) of the primary site was determined by using CE‐CT, FDG‐PET/CT, and a barium study, and was marked with clips at the cranial and caudal ends of the tumor using fiberoptic endoscopy. The GTV of the lymph node metastasis was determined using CE‐CT and FDG‐PET/CT. The clinical target volume (CTV) was defined as the GTV plus a 3–5‐mm margin in all directions. The planning target volume (PTV) was defined as the CTV plus a setup margin of 5 mm and an internal margin (IM) of 2–5 mm. The IM was determined by the stability of respiration under a respiratory gating system (AZ‐733; Anzai Medical, Tokyo, Japan), which was used for beam irradiation during the exhalation phase. A customized vacuum‐lock bag was used for patient immobilization. The total dose at the isocenter was prescribed to cover 90% of the PTV. Doses were calculated using the pencil beam algorithm. Dose constraints were total lung volume receiving greater than 20 Gy (V20) <35% (ideally, <20%), mean lung dose <20 Gy, heart V40 < 40%, liver V30 < 30%, and spinal cord dose <45 Gy. To reduce the dose of cardiac radiation, proton beams were delivered with posterior and posterior oblique angles (Fig. 2).

### Chemotherapy

Chemotherapy regimens consisted of nedaplatin (NDP) and 5‐fluorouracil (5‐FU). Each course of chemotherapy was given prior to radiotherapy and consisted of continuous intravenous administration of 5‐FU at a dose of 700 mg/m^2^/24 h for 5 days (days 1–5) followed by NDP at a dose of 130 mg/m^2^/5 h on day 6. If a patient's serum creatinine level was greater than 1.5 mg/dL on the scheduled date of chemotherapy, the patient did not receive chemotherapy. In addition, if the WBC count was below 3000/mm^2^ or the platelet count was below 75,000/mm^2^, chemotherapy was postponed, and radiotherapy was performed instead. Moreover, if hematological data obtained 2 weeks after radiotherapy did not meet the inclusion criteria (WBC count >3000/mm^2^ and platelet count >75,000/mm^2^), chemotherapy was discontinued. Furthermore, if the WBC count decreased below 1000/mm^2^ or if the platelet count decreased below 25,000/mm^2^ after chemotherapy, the doses of 5‐FU and NDP were reduced by 25% at the next administration.

### Patient assessments

For this analysis, all tumors were staged according to the UICC TNM Classification, 7th edition. According to the Common Terminology Criteria for Adverse Events version 4.0, the toxicity of the ACRT was evaluated by changes in WBC count, neutrophil count, platelet count, hemoglobin level, liver function, renal function, and changes in the esophageal mucosa. The antitumor effects (primary effects) of the ACRT were evaluated based on the results of gastrointestinal fibroscopy and PET‐CT performed 3–4 months after the completion of treatment according to the WHO criteria. Disease progression after treatment was evaluated every 3 months for the first year and every 4–6 months thereafter.

The survival period was measured starting from the day of initiation of ACRT. OS was measured from the first day of treatment until the date of death from any cause. PFS was calculated up to the day of confirmation of tumor growth, detection of a new lesion, or death from other diseases despite tumor control.

### Statistical analysis

Continuous variables are summarized as medians and ranges, and categorical variables as frequencies and proportions. OS, PFS, and LC curves were estimated using the Kaplan–Meier method. The log‐rank test was used in the univariate analysis to evaluate the effect of each of the following prognostic factors on OS: age, gender, T classification, N classification, stage, total radiation dose to the tumor, passage of the GIF before therapy, and tumor response. Due to the small number of events (only 17 in 47 patients), no simultaneous multivariate analysis was performed to adjust for the effects of prognostic factors. These results are summarized as hazard ratios (HRs) and their 95% confidence intervals (CIs) based on the Cox proportional hazards model. All *P*‐values were two sided, and values of *P* < 0.05 were considered to indicate statistical significance. Statistical analyses were performed using R statistical software (A language and environment for statistical computing. R Foundation for Statistical Computing, Vienna, Austria), version 2.15.2.

## Results

### Patient characteristics

Between January 2009 and December 2012, a total of 47 patients (37 male and 10 female) with esophageal cancer were enrolled in this study. Table 1 shows the clinicopathologic characteristics of the subjects. Patient age ranged from 47 to 77 years (median 63 years). Six patients had an ECOG PS of 0, 39 patients had a PS of 1, and two patients had a PS of 2. Histology revealed that 46 cases were squamous cell carcinoma, and one case was adenocarcinoma. Twelve of the 47 (25.5%) patients had inoperable cancer, and the remaining 35 (74.5%) patients had refused surgery.

**Table 1:** Patient characteristics

No. of patients	47
Age (years)	
Median	63
Range	47–77
Sex	
Male	37 (78.7%)
Female	10 (21.3%)
ECOG performance status	
0	6 (12.8%)
1	39 (83.0%)
2	2 (4.3%)
Histology	
Squamous cell carcinoma	46 (97.9%)
Adenocarcinoma	1 (2.1%)
Location	
Upper thoracic	10 (21.3%)
Middle thoracic	19 (40.4%)
Lower thoracic	17 (36.2%)
Abdominal esophagus	1 (2.1%)
Clinical stage (UICC 2009)	
IA	10 (21.3%)
IB	0 (0%)
IIA	3 (6.4%)
IIB	9 (19.1%)
IIIA	15 (31.9%)
IIIB	1 (2.1%)
IIIC	9 (19.1%)

The initial irradiation field included the lymph node regions for prophylactic purposes, and the total X‐ray dose ranged from 12.6 to 40 Gy (median 36 Gy). The proton beam dose was delivered to the entire tumorous lesion and ranged 28.6–63.8 GyE (median 37.4 GyE). The total dose (XRT and PBT) ranged 64.6–80.0 Gy (median 73.4 Gy). The radiotherapy treatment protocol was terminated in one case after 12.6 Gy of XRT because the patient refused further treatment. For this patient, treatment was changed to PBT and a supplemental dose of 63.8 GyE was given, although this did not comply with the protocol requirement. The remaining 46 patients fully completed the treatment protocol. Chemotherapy treatment was discontinued after completion of the first course for four of the 47 patients, and a second course of chemotherapy was not implemented. Of these four patients, one had an allergic reaction, one suffered myelosuppression, and two refused further chemotherapy. Follow‐up studies were performed for all 47 patients at the end of May 2014.

### Acute toxicity

The toxicities associated with CRT are summarized in Table 2. Grade 3–4 leukopenia, neutropenia, anemia, and thrombocytopenia were observed in 26 (55.3%), 21 (44.7%), 2 (4.3%), and 13 (27.7%) of the patients, respectively. Although grade 3–4 acute esophagitis was observed in 5 (10.6%) patients, none developed into pneumonia.

**Table 2:** Acute and late toxicities (according to the Common Terminology Criteria for Adverse Events version 4.0)

No. of patients	47
Acute toxicity (Grade 3 and higher)	
Hematologic	
Leukopenia	26 (55.3%)
Neutropenia	21 (44.7%)
Anemia	2 (4.3%)
Thrombocytopenia	13 (27.7%)
Nonhematologic	
Nausea and vomiting	1 (2.1%)
Esophagitis	5 (10.6%)
Pneumonitis	0 (0%)

Late toxicity (Grade 2 and higher)			
	G2	G3	G4
Pericarditis	0 (0%)	0 (0%)	0 (0%)
Pericardial effusion	9 (19.1%)	0 (0%)	0 (0%)
Pleural effusion	1 (2.1%)	0 (0%)	0 (0%)
Pneumonitis	1 (2.1%)	1 (2.1%)	0 (0%)
Esophageal stenosis	1 (2.1%)	2 (4.3%)	0 (0%)
Esophageal fistula	0 (0%)	1 (2.1%)	0 (0%)

### Late toxicity

Regarding grade 3–4 late toxicities, there were no incidences of pleural and pericardial effusion, but two patients (4.3%) suffered esophageal stenosis, one patient (2.1%) developed a fistula, and two patients (4.3%) developed radiation pneumonitis. The patient who suffered a grade 3 fistula had received a 75.6 Gy dose to the lesion in the esophagus. Two patients died of possible treatment‐related toxicity at 3 and 10 months after completion of treatment. One patient developed radiation pneumonitis in association with a connective tissue disorder after ACRT; steroid therapy was provided for this patient, but he died of *Pneumocystis carinii* pneumonia after 10 months. Another patient with grade 3 esophagitis and pneumonia died of an unknown cause after 6 months.

### Response

Of the 47 patients with stage I–III, 37 (78.7%) had a complete response (CR), and 10 (21.3%) had a partial response (PR). The local failure occurred in 11 of the 47 patients (23.4%), the recurrence at the prophylactic area of lymph nodes occurred in two of the 47 patients (4.3%), and the distant metastasis occurred in five of the 47 patients (10.6%).

Local failure occurred in nine of the 29 patients (31.0%) with stage II/III cancer. Eight of these nine patients (88.9%) involved a PBT irradiation field, while in the remaining one patient, there was an XRT irradiation field outside of the PBT field. Local treatment failure occurred at the primary site in seven of the nine patients (77.8%) and in the lymph nodes in two of the nine patients (22.2%).

### Survival

**Figure 3:**
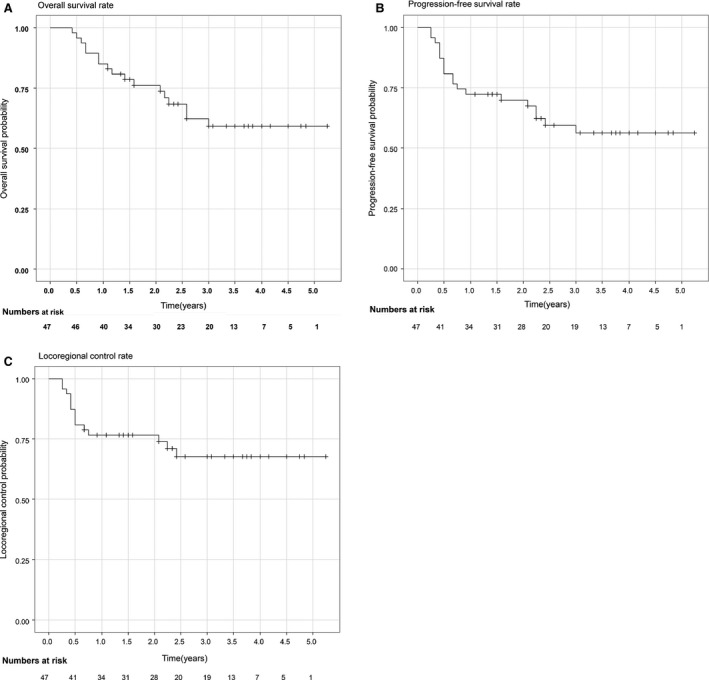
Overall survival (OS), progression‐free survival (PFS) and locoregional control (LC) in 47 patients analyzed by the Kaplan–Meier method.

**Figure 4:**
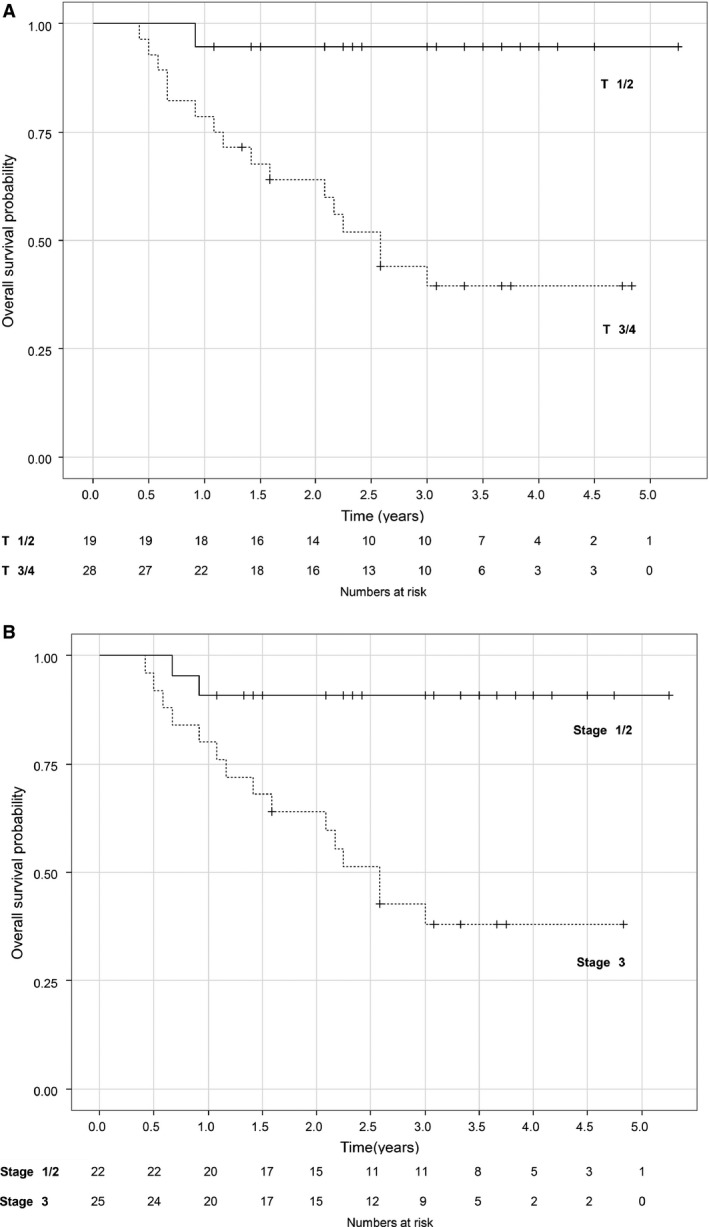
Overall survival (OS) stratified by T factor(4a) and Stage(4b).

The median follow‐up duration was 29 months (range 5–63 months) for all patients, and 40 months (range 13–63 months) for survivors. At the time of survival analysis in May 2014, 30 (68.8%) patients were alive, and 28 (93.3%) of these 30 surviving patients remained free of cancer. The 3‐year OS, PFS, and LC rates were 59.2% (95% CI, 45.7–76.8%), 56.3% (95% CI, 43.0–73.7%), and 67.7% (95% CI, 54.9–83.6%), respectively (Fig. 3). The rate of therapy‐related death was 4.3% (2/47). Table 3 shows the 3‐year survival rates and their associated *P*‐values calculated by univariate analyses of various factors that may affect prognosis. By univariate analysis, we identified four possible factors influencing survival rate: T status (T1–2 vs. T3–4), primary effect (CR vs. non‐CR), endoscopic finding (bounded vs. unbounded), and the passage of a GIF prior to initiation of therapy (yes vs. no). The 3‐year OS rate for patients with T1–2 and T3–4 lesions was 94.7% (95% CI, 85.2–100%) and 39.6% (95% CI, 24.5–63.9%), respectively, and for patients with stage I/II and stage III disease was 90.9% (95% CI, 79.7–100%) and 37.9% (95% CI, 22.6–63.6%), respectively (Fig. 4).

**Table 3:** The 3‐year survival rates and their associated *P*‐values calculated using univariate analyses of various factors

Factor	Level	*n*	Univariate analysis (Log‐rank test)		
			HR	95% CI	*P*‐value
Gender	Female (referent)	10			
	Male	37	2.389	0.545, 10.474	0.231
Age (year)	<63 (referent)	21			
	≥63	26	1.013	0.390, 2.627	0.979
T stage	1, 2 (referent)	19			
	3, 4	28	12.975	1.720, 97.893	0.001
N stage	0 (referent)	14			
	1, 2, 3	33	3.657	0.835, 16.010	0.064
Stage	1, 2 (referent)	22			
	3	25	7.392	1.690, 32.340	0.002
Location	Ut‐Mt (referent)	29			
	Lt‐Ae	18	1.316	0.507, 3.415	0.570
Total dose (Gy)	≤70 (referent)	20			
	>70	27	2.672	0.869, 8.210	0.073
Primary effect	Non‐CR (referent)	10			
	CR	37	0.108	0.040, 0.287	<0.001
Finding of GIF	Clear margin (referent)	34			
	No clear margin	13	2.805	1.075, 7.320	0.027
Passage of GIF	Yes (referent)	40			
	No	7	6.903	2.495, 19.097	<0.001
SUV max	<14.5 (referent)	23			
	≥14.5	22	3.434	1.093, 10.795	0.024

CI, confidence interval; GIF, gastrointestinal fiberscope; CR, complete response; SUV, standardized uptake value; Ut‐Mt, Upper thoracic esophagus‐ Middle thoracic esophagus; Lt‐Ae, Lower thoracic esophagus ‐Abdominal esophagus.

Twenty‐nine patients in this study had stage II/III (non‐T4) cancer, and the median follow‐up period was 41 months. Twenty‐four of these patients (82.8%) achieved a CR. The 3‐year OS, PFS, and LC rates were 54.8% (95% CI, 37.9–79.4%), 54.4% (95% CI, 38.0–78.0%), and 69.8% (95% CI, 54.0–90.2%), respectively.

## Discussion

Recently, many authors have reported promising results with CCRT for esophageal cancer. However, serious late adverse effects and a local recurrence rate of approximately 50% after CCRT are important problems 6, 8. In order to improve the LC rate and reduce adverse effects, we used PBT combined with ACRT.

In our study, XRT was initially used for the extended field irradiation. This method was chosen because squamous cell carcinoma of the esophagus has a high potential for widespread lymph node metastasis. It has been reported that 42% of patients with stage T1‐3N0M0 esophageal cancer had pathological lymph node metastases 17. Even when lymph node metastasis is not detected using radiological images prior to treatment, it is still necessary to irradiate a wider field. Because the available PBT field size in our facility is 15 × 15 cm, we used XRT for the extended field.

The most effective way to combine chemotherapy with radiotherapy is concurrently. However, CCRT has been associated with increased acute toxicity and a decreased compliance rate 8, 18. Favorable results using ACRT have been reported for head and neck cancer and esophageal cancer 19, 20. According to the Intergroup 0099 study (IGS) report, 56% of patient with nasopharyngeal carcinoma developed distant metastasis after CRT due to insufficient chemotherapy doses 21. Meanwhile, Goto et al. reported promising results and a high compliance rate when ACRT was used to treat 100 patients with advanced nasopharyngeal carcinoma. The 5‐year rate of distant metastasis‐free survival (DMFS) was 87.8% 22. ACRT may be a useful method for reducing distant micrometastasis because a sufficient amount of anticancer agents can be administered, and there is a low frequency of acute adverse effects.

We selected NDP (254‐S), which, compared to cisplatin, causes fewer adverse effects such as nausea, vomiting, appetite loss, and renal dysfunction 23, 24. Jingu et al. reported the results of a phase II study on the treatment of postoperative locoregional recurrent esophageal cancer with radiotherapy combined with chemotherapy consisting of 2 cycles of NDP and 5‐FU 24. The 1‐year and 3‐year OS rates were 60.6% and 56.3%, respectively, with a median survival of 39.0 months. Radiotherapy combined with NDP and 5‐FU is a safe and effective treatment for postoperative locoregional recurrent esophageal cancer.

It has been reported that the OS for patients with stage II/III (non‐T4) esophageal squamous cell carcinoma (ESCC) treated with CRT was 42–45% at 3 years and 29–37% at 5 years 6, 7. Similarly, it has been reported that the 3‐year and 5‐year OS for patients in Japan with stage II/III (non‐T4) ESCC treated with radical surgery were 40–63% and 40–55%, respectively 18, 25. Trimodal therapy is the current standard of care for the management of non‐metastatic esophageal cancer in Europe and the United States. In a randomized phase III trial comparing preoperative CRT to surgery alone, the superiority of preoperative CRT in OS was demonstrated, with 3‐year and 5‐year OS rates of 58% and 47%, respectively 26. To date, our results, which showed 3‐year OS, PFS, and LC rates of 54.8%, 54.4%, and 67.7%, respectively, for stage II/III (non‐T4) cancer and 59.2%, 56.3%, and 69.8%, respectively, for stage I–III cancer, were superior to results with conventional CRT and nearly equivalent to results with surgery and trimodal therapy.

Previous studies of CRT reported a grade 3 or higher cardiac and pulmonary complication rate of around 10% and a treatment‐related death rate of 2.6–5.0% 8, 11. Cardiac disturbance may result from a disorder of the vascular endothelial cells or microvessels after RT, which could lead to continued pericarditis, pericardial effusion, cardiac myopathy, valvular disorder, or ischemic cardiac disease 27. Morota et al. studied late adverse events after CRT for esophageal cancer and found that patients aged 75 years or older were at greater risk of cardiopulmonary toxicity 28. In our study, nine patients (19.1%) had grade 2 cardiac and pleural effusions, but none had cardiac complications of grade 3 or worse, and only two patients (4.3%) were affected by grade 3 or higher pulmonary complications. This may have been because patients in this study were relatively young (median age 63 years, range 44–77 years) and had no history of serious cardiopulmonary complications. Furthermore, boost PBT irradiation from the rear could reduce the dose to the coronary artery and might therefore reduce cardiac disturbance. Our findings suggest that PBT irradiation carries a lower risk of late heart and lung toxicity.

There have been a few reports of the use of PBT for esophageal cancer. Sugahara et al. reported the results of a clinical phase I study of PBT with or without XRT for 46 patients 15. Forty patients received a combination of XRT (median, 48 Gy) and PBT (median, 31.7 Gy) as a boost. The remaining six patients received only PBT (median, 82.0 Gy; range, 75–89.5 Gy). Chemotherapy was not used. The 5‐year actuarial survival rate was 34%. Eight percent of patients had grade 3 late esophageal complications, and 5% of patients had grade 5 esophageal complications. No symptomatic late complications were observed in the tracheobronchial trees or heart. Lin et al. reported a retrospective analysis of a prospective study evaluating normal tissue toxicity from concurrent chemotherapy and PBT (CChT/PBT) 16. A total of 29 (46.8%) patients received preoperative CChT/PBT, and 33 (53.2%) patients received definitive CChT/PBT. The CR rate and the 3‐year OS, LC, and PFS were 50%, 51.7%, 56.5%, and 40.5% respectively. There were two cases of grade 2 and 3 radiation pneumonitis and two cases of grade 5 radiation pneumonitis. The results of this study might be equivalent to or better than those of the other two PBT studies. Our results might be closely related to the use of extended field irradiation, high doses of chemotherapy, and a high total dose of radiotherapy for the primary lesion and lymph node metastases.

Univariate analysis in this study showed that the ability to pass an 8.9‐mm diameter GIF through the esophagus before treatment is an independent prognostic factor for OS. An obstruction in the passage of a GIF indicates muscle invasion that spreads into the entire wall of the esophagus. The ability to pass a GIF might be one of the objective criteria to identify the tumor as a candidate for PBT instead of surgery.

Our study has a number of limitations, including its retrospective design, the inclusion of only a small number of patients, the use of NDP‐based chemotherapy, and a short observation period. Despite this, our findings indicate that the use of PBT with ACRT, which increases the radiation dose to the tumor while minimizing the exposure to the heart and lungs, can improve the CR rate and long‐term prognosis of esophageal cancer patients. Further research is therefore warranted to more fully define its clinical role.

## Conclusion

Although a longer observation period is required for a definitive conclusion, PBT with ACRT might be able to effectively treat the tumor and its associated metastases. PBT will play an important clinical role as a therapeutic tool for patients with esophageal cancer and may eventually become one of the primary methods of treatment of this disease.

## Conflict of Interest

The authors have no conflict to disclose.
